# A binder-free electrode architecture design for lithium–sulfur batteries: a review

**DOI:** 10.1039/c9na00040b

**Published:** 2019-04-25

**Authors:** Junling Guo, Jinping Liu

**Affiliations:** School of Chemistry, Chemical Engineering and Life Science, State Key Laboratory of Advanced Technology for Materials Synthesis and Processing, Wuhan University of Technology Wuhan 430070 People's Republic of China liujp@whut.edu.cn; State Center for International Cooperation on Designer Low-carbon & Environmental Materials, Zhengzhou University 100 Kexue Avenue Zhengzhou 450001 People's Republic of China

## Abstract

Lithium–sulfur batteries (LSBs) are considered to be one of the most promising next-generation electrochemical power sources to replace commercial lithium-ion batteries because of their high energy density. However, practical application of LSBs is hindered by two critical drawbacks: “redox shuttle reactions” of dissolved polysulfides at the cathode side and Li dendrites at the Li anode side. Therefore, various approaches have been proposed to break down technical barriers in LSB systems. The overall device performance of LSBs depends on not only the development of host materials but also the superior architecture design of electrodes. Among these architectures, binder-free electrodes are verified to be one of the most effective structural designs for high-performance LSBs. Therefore, it is urgent to review recent advances in binder-free electrodes for promoting the fundamental and technical advancements of LSBs. Herein, recently emergent studies using various binder-free architectures in sulfur cathodes and lithium metal anodes are reviewed. These binder-free electrodes, with well-interconnected structures and abundant structural space, can provide a continuous pathway for fast/uniform electron transport/distribution, load sufficient active materials for ensuring high energy density, and afford large electrochemically active surface areas where electrons and Li ions can come into contact with the active materials for fast conversion reactions, thus leading to suitable applications for LSBs. Subsequently, the advantages and challenges of binder-free architectures are discussed from several recently emergent studies using binder-free structured sulfur cathodes or Li metal anodes. The future prospects of LSBs with binder-free electrode structure designs are also discussed.

## Abstract 2

Binder-free structures offering structural and kinetic advantages for constructing high performance sulfur cathodes and dendrite-free Li metal anodes are reviewed.

## Graphical abstract

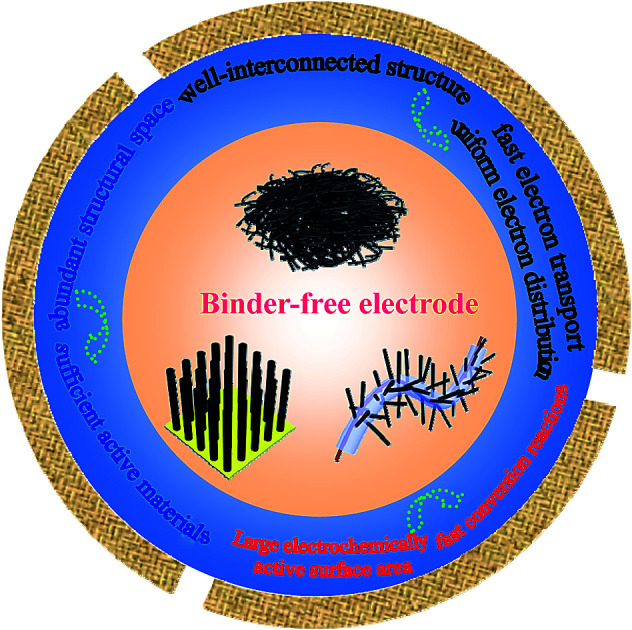

## 1. Introduction

**Fig. 1:**
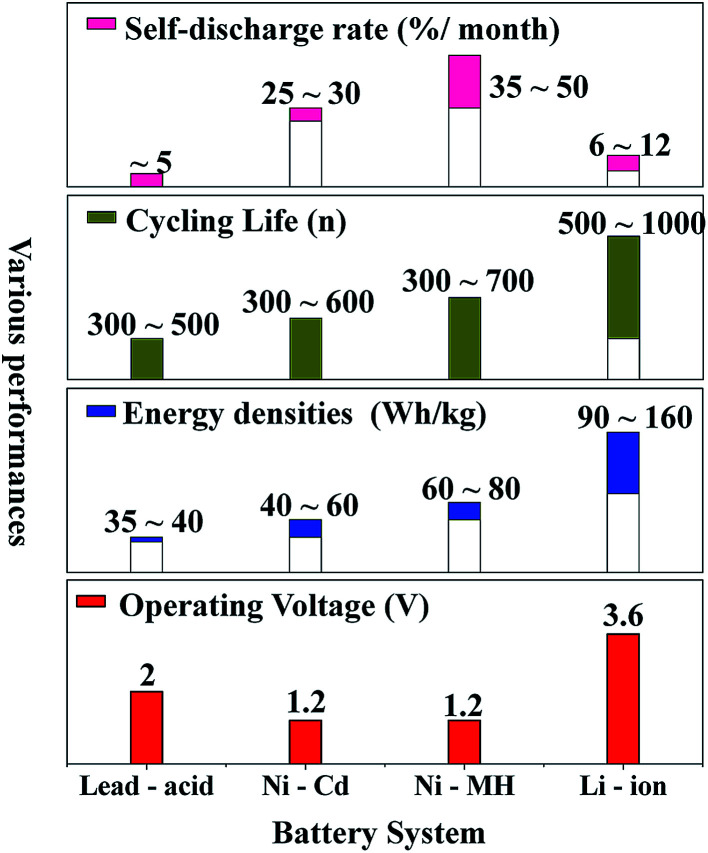
The performance comparison of various commercialized batteries.

Nowadays, among commercialized devices for electrical energy storage, lithium ion batteries (LIBs) are the predominant power sources for wide applications due to their high working potential, large energy/power density, long cycle life and environmental friendliness (Fig. 1).^1–3^ However, the energy density of LIBs gradually approaches the theoretical limit, but is still unable to meet the ever-increasing energy density demand for many future applications, such as electric vehicles, large-scale electrical grids, satellites and so on.^4–6^ Alternatively, lithium–sulfur batteries (LSBs) with high theoretical energy densities have attracted considerable attention of the whole world.^7–9^

**Fig. 2:**
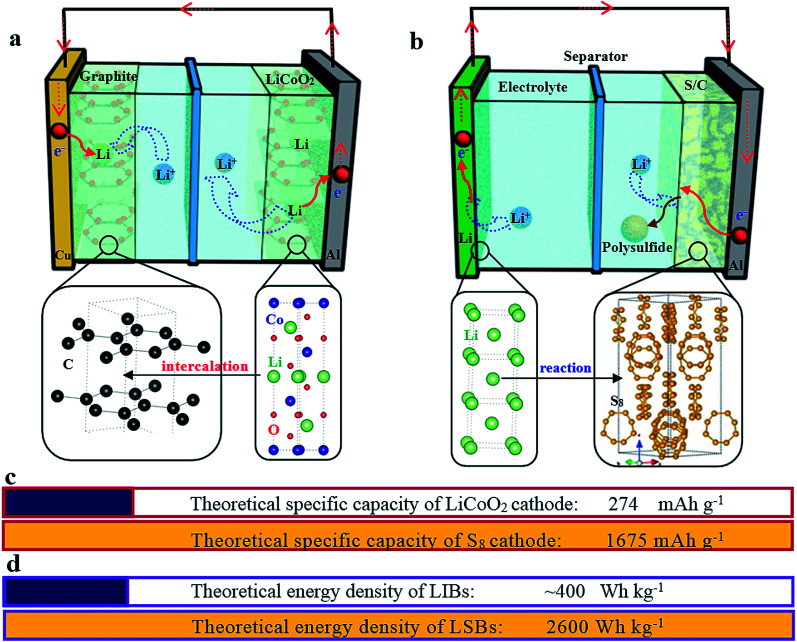
A schematic illustration of the working principles of a graphite/LiCoO_2_ lithium-ion battery (a) and a lithium–sulfur battery (b). The crystal structure of graphite, LiCoO_2_, Li metal and sulfur is also given at the corresponding positions. (c) The comparison of theoretical specific capacities of the LiCoO_2_ and S_8_ cathodes. (d) The comparison of theoretical energy densities of LIBs and LSBs.

**Fig. 3:**
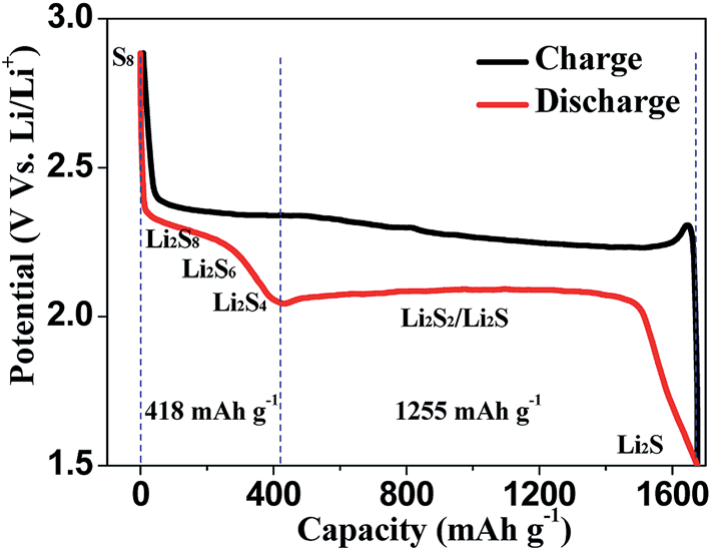
The typical charge–discharge curve of lithium–sulfur batteries.

Different from the intercalation/deintercalation electrochemical reaction of LIBs displayed in Fig. 2a,^10,11^ the prototype of LSBs is based on an overall reaction between sulfur and Li metal of S_8_ + 16 Li → 8 Li_2_S (Fig. 2b). This reaction gives rise to a high theoretical specific capacity of sulfur cathodes (1675 mA h g^−1^), which is much larger than that of LiCoO_2_ (Fig. 2c). Therefore, the energy density of LSBs (2600 W h kg^−1^) is ∼5 times higher than that of conventional LIBs. However, this reaction also leads to some disadvantages impeding the practical application of LSBs.^12–14^ Since the as-written overall reaction is too simple to find the reasons causing these drawbacks, many efforts have been devoted to exploring the actual detailed reaction process of LSBs. To date, a complicated and intermediate reaction has been widely accepted by researchers.^15–17^ As Fig. 3 shows, in the early discharge process, S_8_ obtains Li-ions and electrons to form a series of long-chain lithium polysulfide species (S_8_ → Li_2_S_8_ → Li_2_S_6_/Li_2_S_4_). Since this process involves multiple reactions which obtain just 4 electrons per S_8_, the corresponding voltage plateau is sloping and contributes just 25% of the theoretical capacity of sulfur (418 mA h g^−1^). In the subsequent discharge process, Li_2_S_4_ is further lithiated and forms short-chain sulfide species (Li_2_S_4_ → Li_2_S_2_/Li_2_S). Because this process involves an individual reaction which obtains 12 electrons per S_8_, the corresponding voltage plateau is flat and contributes to 75% of the theoretical capacity of sulfur (1255 mA h g^−1^).^18^

**Fig. 4:**
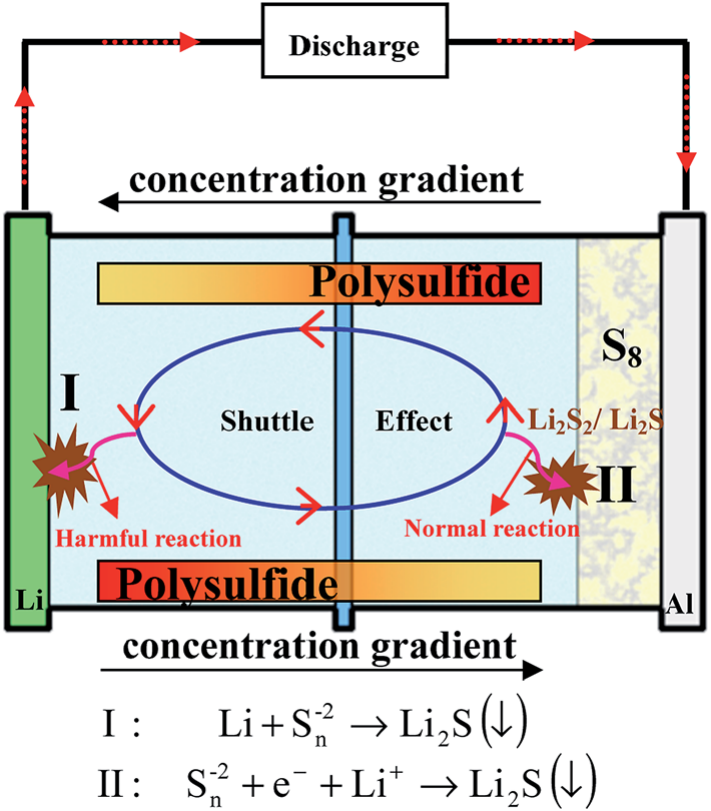
Illustration of the well-known shuttle effect caused by the dissolution of polysulfides.

From the above-mentioned details of the reaction process, the challenges impeding the practical application of sulfur cathodes can be discussed,^19–21^ including:

(1) “Shuttle effect”: the intermediate long-chain lithium polysulfide species dissolve instantly into electrolytes. Thereby, the dissolved polysulfide species can diffuse from the cathode to the lithium anode and then return to the sulfur cathode with the change of the concentration gradient, leading to the well-known shuttle effect (Fig. 4). During this diffusion, the soluble polysulfide species can be directly reduced by Li metal without electrons present to form short-chain polysulfides (Li_2_S_2_/Li_2_S). Then these insoluble Li_2_S_2_/Li_2_S will be deposited on Li metal and cannot be used in the following cycles, thus giving rise to a severe loss of active materials of the cathode, and high self-discharge and rapid capacity decay of the sulfur cathode. However, it should be noted that the soluble polysulfide can ensure the fast kinetics of this process (S_8_ → Li_2_S_8_ → Li_2_S_6_/Li_2_S_4_), leading to a high utilization of sulfur. This is because no insulating layer is formed on the surface of the conductive material with the dissolution of insulating polysulfide, guaranteeing the electron conductivity of the cathode in this process.

(2) “Low utilization of sulfur”: the insulating nature of sulfur and lithium sulfide results in a very low utilization of sulfur, especially during the process Li_2_S_4_ → Li_2_S_2_/Li_2_S providing the major capacity of Li–S batteries. The generated insoluble and insulating Li_2_S_2_ or Li_2_S will be deposited on the surface of the conductive material, thus making the reaction kinetics slow, increasing the polarization and leading to inferior utilization of sulfur and poor rate performance.

(3) “Unstable cathode structure”: since the density of sulfur (S_8_) and lithium sulfide (Li_2_S) is different (2.03 *vs.* 1.66 g cm^−3^, respectively), there is a huge volume fluctuation (∼80%) of sulfur during the discharge/charge process, which leads to an unstable cathode structure. Therefore, the cycle performance of sulfur cathodes is not only affected by the shuttle effect but also by the volume expansion.

**Fig. 5:**
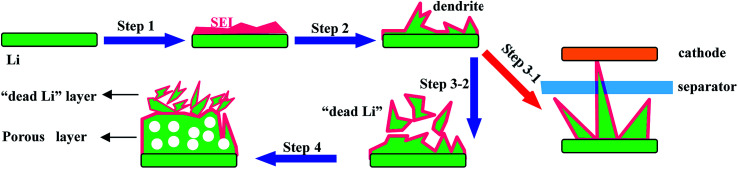
A schematic illustration of the Li stripping/plating process. Step 1 is the formation of the SEI; step 2 is the formation of dendrites; step 3-1 is the fracture of the separator; step 3-2 is the formation of “dead Li” and step 4 is the formation of the porous structure.

The reaction of sulfur also suggests that S_8_ obtains electrons during the discharge process,^7^ which is almost similar to that of anode materials (C, Si, *etc.*) of LIBs, indicating that it is necessary to use Li metal as the anode in LSBs.^12^ Therefore, the challenges of a Li metal anode also hinder the practical application of LSBs. These issues of metallic Li anodes are caused by Li dendrites (Fig. 5), which are inevitably generated during the charge/discharge process due to repetitive Li plating/stripping at the electrode surface.^22–24^ These drawbacks are listed as follows:

(1) “Low coulombic efficiency”: Li metal can react with the electrolyte directly to form a passivating film on the Li metal/electrolyte interface known as the solid electrolyte interface (SEI); note that the SEI can allow Li-ion transmission but does not permit electron transport (step 1 in Fig. 5). However, the SEI is easily cracked because of the huge volume expansion caused by the formation of Li dendrites (step 2 in Fig. 5). Consequently, this SEI layer is unable to protect the Li metal anode from the undesirable and irreversible reactions with the electrolyte, leading to continuous consumption of both Li metal and the electrolyte, thus giving rise to poor reversibility and low coulombic efficiency.

(2) “Low safety”: with the unceasing growth of Li dendrites, the separator may be penetrated by dendrites (step 3-1 in Fig. 5), resulting in internal short circuits and then leading to serious safety hazards.

(3) “Low utilization of Li”: some dendrites may be fractured during their growing process and then will be completely encapsulated by the SEI, leading to non-contact with the main anode. This part of Li metal is well-known as “dead lithium” since it is unable to be utilized in the following cycling processes (step 3-2 in Fig. 5), leading to low utilization of Li.

(4) “Large polarization”: the Li metal anode always forms porous and uneven structures during further charge–discharge processes (step 4 in Fig. 5), which lead to longer diffusion pathways of Li ions and larger resistance of electrons, thus causing a large polarization of the Li metal anode.

To break down the aforementioned technical barriers in LSB systems, various approaches have been proposed in the past 10 years. Rational design to address the challenges with the sulfur cathode should include the following features:^25–27^

(a) A strategy to provide high conductivity of the whole sulfur cathode, resulting in desired sulfur utilization;

(b) A method to confine the shuttle effect of lithium polysulfide species, guaranteeing the long cycle life of the sulfur cathode;

(c) A method to accommodate the volume fluctuation (∼80%) of sulfur during the discharge/charge process, ensuring the structural stability of the cathode.

Nowadays, a typical strategy to obtain high performance sulfur cathodes is the use of various structural host materials (such as carbon materials,^28–31^ polar metal oxides and sulfides,^32–35^ conducting polymers^36–38^ and their composites^39–41^), which could improve the conductivity and confine lithium polysulfide species through physical/chemical effects. These host materials have their own advantages and disadvantages: carbon materials can significantly improve the utilization of sulfur due to their high conductivity, and somewhat enhance cyclability because they can adsorb polysulfide through weakly physical functions. For example, Li and co-workers designed a sulfur cathode *via* using amorphous carbon (AC) as the host material, which exhibited a high reversible capacity of 1220 mA h g^−1^ at 0.1C and an enhanced but unsatisfactory cyclability (68% of the initial capacity retained after 50 cycles);^42^ in contrast, metal oxides/sulfides can further improve the cycle performance of sulfur cathodes because of their polar nature, and can adsorb polysulfide by strong chemical functions, and somewhat enhance the utilization of sulfur due to their conductivity being higher than that of sulfur but much lower than that of carbon materials. For example, Cui *et al.* designed and fabricated a sulphur–TiO_2_ yolk–shell nanoarchitecture with an internal void space to further improve the cycle performance of sulfur cathodes. In this work, the TiO_2_/S cathode retained 67% of the initial capacity after 1000 cycles, displaying an ultra-long cycle life. However, the initial capacity of this cathode is just 1030 mA h g^−1^ at 0.5C, which is lower than that using carbon–sulfur composite materials.^43^

**Fig. 6:**
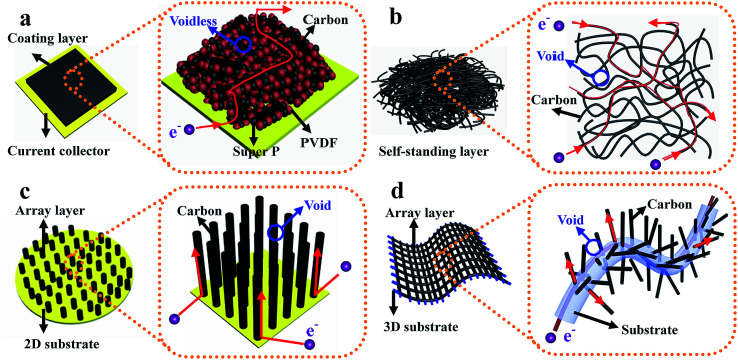
The schematic illustration of the comparison between the electrode with binder and several typical binder-free electrodes. (a) Electrode with binder; (b) self-standing electrode; (c) array structure on a 2D substrate electrode; (d) array structure on a 3D substrate electrode.

Rational design to address the challenges on the Li anode suppresses dendrite growth effectively. Up to now, there are four typical methods to obtain the high performance Li anode, including:

(a) LiX (X = Al, B, Si, Sn, C, *etc.*) alloy strategy: this method can suppress the dendrites by storing lithium in the ionic form.^44,45^ However, its application is limited by the large volume changes during cycling of the LiX alloy (X = Al, B, Si, Sn) or drastically sacrificed capacity of the LiX alloy (X = C);

(b) Li metal/electrolyte interface modification strategy: this method can protect the lithium metal *via* forming a passivating film on the Li metal/electrolyte interface.^46,47^ Unfortunately, this passivating film is breakable due to the large volume changes caused by Li dendrites. Therefore, there is an imperative requirement for design of passivating films with high shear modulus or flexibility;

(c) Solid-state electrolyte strategy: this strategy can impede the formation of dendrites because of the high shear modulus of solid-state electrolytes (about twice that of the Li dendrites).^48,49^ However, the ionic conductivity of solid-state electrolytes (10^−8^ and 10^−5^ S cm^−1^ at room temperature) is typically lower than that of commercial liquid electrolytes (10^−3^ S cm^−1^), restricting the application of solid-state electrolytes.

(d) Structured anode design strategy: dendrite-free Li metal anodes can also be achieved by using novel architectures. For instance, Zheng *et al.* designed interconnected hollow carbon nanospheres on Cu foil to protect the fragile SEI layer, thus leading to dendrite-free anodes.^50^ Yan and co-authors regulated lithium metal to selectively deposit into hollow carbon spheres (HCS) through the Au nanoparticles loaded inside HCS, which showed no nucleation barriers for Li deposition. Such selective deposition and stable encapsulation of lithium metal eliminated dendrite formation.^51^ Liang *et al.* demonstrated that a 3D polymer nanofiber structure was capable of homogenizing the distribution of electrons/thermodynamics, thus hindering dendrite formation.^52^ The above discussion suggests that effective improvement of the overall device performance of LSBs can be realized by various superior architecture designs of electrodes. Among these architectures, binder-free electrode structures are verified to be one of the most effective strategies because they have some characteristics of this type of structure which are different from those of electrodes with binders.^53,54^ As shown in Fig. 6a, for traditional powder electrodes, the insulating binder particles (such as polyvinylidene fluoride, sodium alginate, and so on) will impede the continuous transmission path of electrons, especially in powder electrodes with insulating active materials (for example sulfur). Conversely, in binder-free electrodes (such as self-standing films, array structures on different substrates, *etc.* displayed in Fig. 6b–d): (1) the interlinked architecture not only shortens transport distances for ions in the well-interconnected structure, but also provides a continuous pathway for fast electron transport, thus ensuring the fast kinetics of these binder-free electrodes; (2) the abundant structural space can ensure that this structure loads sufficient active materials and that there is adequate contact between active materials and the electrolyte, leading to a high-performance electrode with large areal loading of active materials; (3) this structure provides a large electrochemically active surface area where electrons and Li ions can come into contact with the active materials for fast conversion reactions due to the continuous pathways and abundant exposed surface area, leading to high utilization of active materials; (4) abundant structural voids between neighboring nanostructures can provide enough space to accommodate the significant volume changes of active materials during the charge–discharge process. Therefore, it is urgent to review recent advances in this direction to promote the fundamental and technical advancements of LSBs.

In this review, we aim to provide a comprehensive review of recent advancements in the field of LSBs with binder-free electrode structures. First, we present the recent advances in sulfur cathodes by using binder-free structures and point out advantages and challenges (Section 1). Next, we review the recently proposed strategies to suppress dendrite growth *via* using binder-free structures and highlight the functions of these structures (Section 2). We end the review with an outlook and proposal of several possible improvement trends to further design the binder-free structured electrodes for LSBs.

## 2. Binder-free structure for sulfur cathodes

### 2.1 Self-standing film

Self-standing films with large inter-spaces and low manufacturing costs have some advantages for sulfur cathodes:^55–62^ (1) the effect of insulating sulfur and its insoluble discharge products (Li_2_S_2_/Li_2_S) on electron transport in sulfur cathodes can be mitigated through a continuous pathway for fast electron transport provided by the interlinked architecture; (2) this structure provides large electrochemically active surface areas where electrons and Li ions can come into contact with the sulfur for fast conversion reactions; (3) the abundant structural space accommodates the significant volume changes of sulfur during the charge/discharge process. Therefore, the sulfur cathode using host materials with a binder-free film structure exhibits superior electrochemical performance.

#### 2.1.1 Three-dimensional carbon network self-standing film

**Fig. 7:**
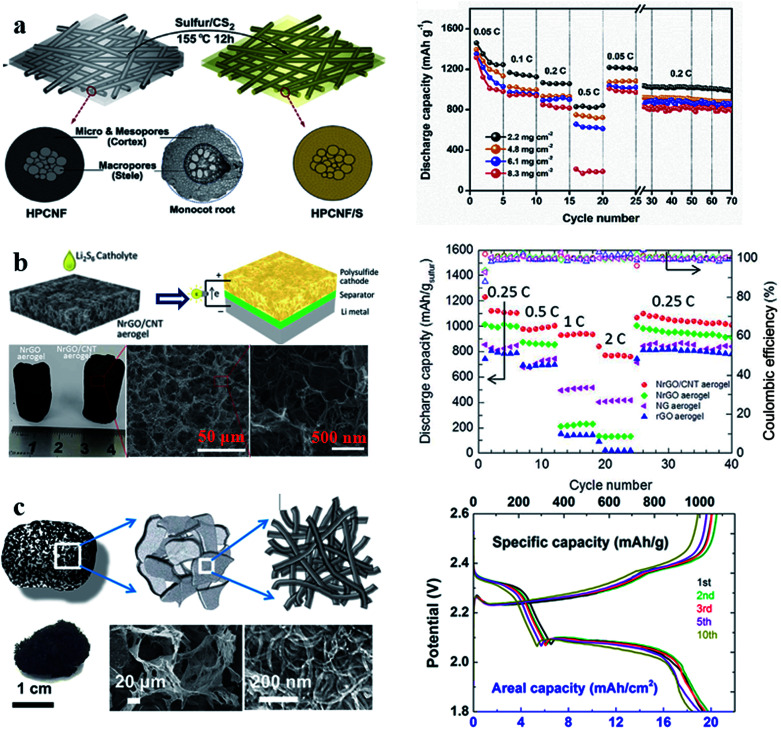
(a) Schematic illustration of the synthesis of the S/HPCNF cathode and its rate performance; (b) schematic illustration and characterization of the sulfur/r-GO/CNT hybrid cathode and its rate performance; (c) schematic illustration and characterization of 3D CNT foam and the galvanostatic charge/discharge curves of the sulfur-3D CNT foam at 0.1C. (a) Reproduced with permission.^68^ Copyright 2018, Elsevier; (b) reproduced with permission.^69^ Copyright 2016, Elsevier; (c) reproduced with permission.^70^ Copyright 2017, American Chemical Society.

By virtue of the advantages of large surface area, light weight, good electrical conductivity, and high thermal/chemical stability, carbon materials have been considered as the most important host materials for sulfur cathodes. In the carbon/sulfur composite cathode, carbon materials can provide ideal reaction sites for sulfur due to their good electrical conductivity, leading to superior sulfur utilization; carbon materials can also adsorb polysulfides using physical interactions because of their large surface area. However, the presence of sulfur on the outer surface of carbon materials can seriously affect the conduction of electrons and the electrode structural stability, especially in a cathode with a high sulfur content. Interlinked 3D carbon nanomaterial network electrodes with the advantages of carbon materials and self-standing film structures can address this issue and thus show outstanding performance in terms of sulfur utilization and reversibility even in a cathode with large sulfur loading.^63–67^ For example, Zhao *et al.* reported a flexible sulfur/hierarchical porous carbon nanofiber (S/HPCNF) binder-free cathode (Fig. 7a) for Li–S batteries, which exhibited a high capacity of 1316 mA h g^−1^ at 0.02C with a large areal sulfur loading (8.3 mg cm^−2^).^68^ Li *et al.* synthesized a 3D reduced graphene oxide/carbon nanotube (r-GO/CNT) hybrid aerogel to improve the performance of the sulfur cathode.^69^ The sulfur/r-GO/CNT cathode reported in this work (Fig. 7b) showed a high capability (767 mA h g^−1^ at 2C) at a high areal sulfur loading weight of 6 mg cm^−2^. Zhao *et al.* prepared a high performance cathode using 3D CNT foam (Fig. 7c). With the advantages of this structure, the prepared cathode with 19.1 mg cm^−2^ areal sulfur loading could deliver a discharge capacity 1039 mA h g^−1^ at 0.1C.^70^ These results suggest that various 3D carbon network films can effectively improve the utilization of sulfur, even in a cathode with large areal sulfur loading, confirming that the advantages of carbon materials and self-supporting structures can indeed realize larger areal sulfur loading, *i.e.* higher energy density. However, the cycle performance of sulfur cathodes is still unsatisfactory because of the weak physical absorption.

**Fig. 8:**
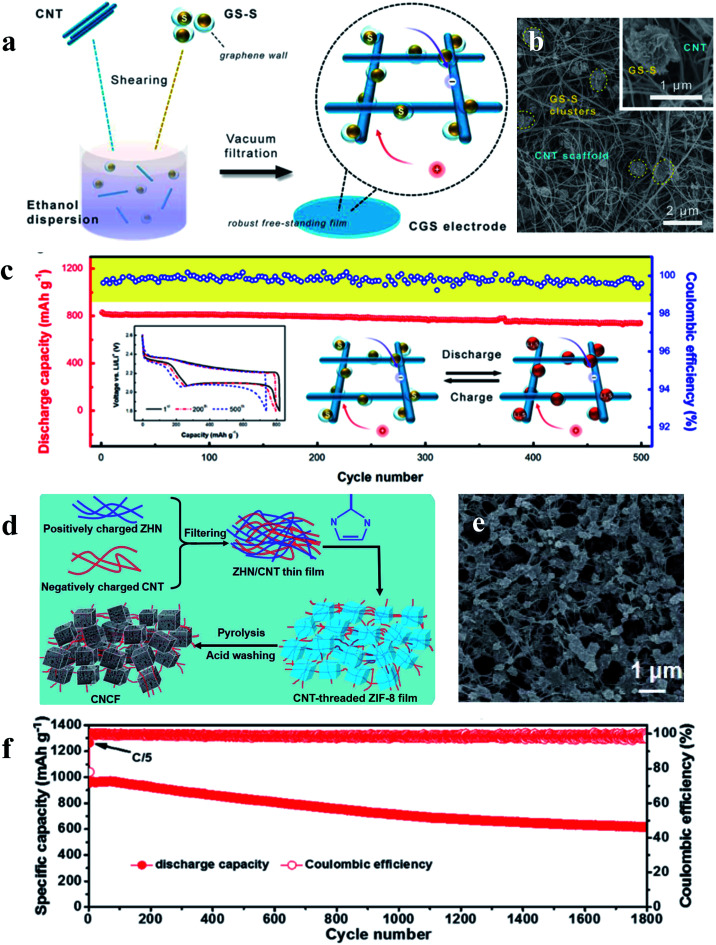
(a–c) Schematic illustration, characterization and the cycle performance (0.5C) of the sulfur cathode with interconnected carbon nanotube/graphene nanosphere scaffolds; (d–f) schematic illustration, characterization and the cycle performance (1C) of the sulfur cathode with CNT-threaded nitrogen-doped porous carbon films. (a–c) Reproduced with permission.^71^ Copyright 2018, Royal Society of Chemistry; (d–f) reproduced with permission.^72^ Copyright 2016, Elsevier.

The performance of sulfur cathodes can be further improved *via* more elaborate structural design. For example, Zhu *et al.* demonstrated that a sulfur cathode with interconnected carbon nanotube/graphene nanosphere scaffolds (Fig. 8a–c) can show a high sulfur utilization of 81% (corresponding to 1346 mA h g^−1^) at a current rate of 0.1C.^71^ Liu *et al.* synthesized a CNT-threaded nitrogen-doped porous carbon film (Fig. 8d–f), which can improve the sulfur cathode capacity to 1057 mA h g^−1^ at 0.2C with a high sulfur-loading of 6.9 mg cm^−2^.^72^

#### 2.1.2 Three-dimensional hybrid material network self-standing film

**Fig. 9:**
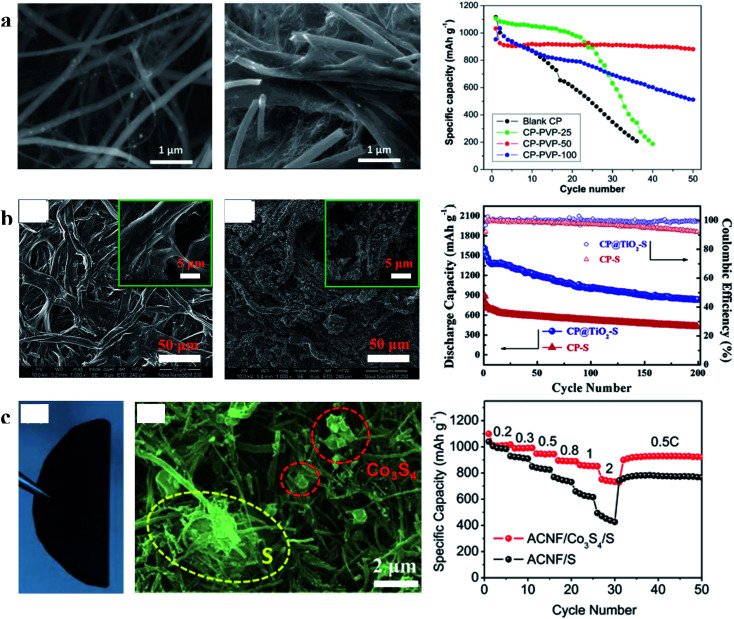
(a) Characterization of CP and CP-PVP and the cyclability of the sulfur cathode with CP and CP-PVP at 0.01C; (b) characterization of CP and CP@TiO_2_ and cycling performances of the sulfur cathode with CP and CP@TiO_2_ at 0.5C; (c) characterization of S/Co_3_S_4_ on the ACNF cathode and its rate performances. (a) Reproduced with permission.^78^ Copyright 2015, American Chemical Society; (b) reproduced with permission.^79^ Copyright 2015, Elsevier; (c) reproduced with permission.^80^ Copyright 2017, Elsevier.

To further enhance the restriction of polysulfide dissolution and improve the cycle performance of carbon/sulfur cathodes, inorganic polar metal oxides/sulfides and conducting polymers are often used to modify these carbon materials.^73,74^ However, this method will affect other properties of the sulfur cathode at the same time. For example, for a sulfur cathode with polar metal oxide modified carbon materials, the cyclability can be improved by polar metal oxides which can trap polysulfide intermediates more efficiently than carbon materials. However, their very low conductivity will seriously injure the sulfur utilization when compared with carbon. Therefore, it is necessary to retard the effect of modifying materials on other properties of the sulfur cathode. The 3D carbon nanomaterial network electrodes with continuous pathways can address this issue significantly. In such modified carbon network electrodes, the 3D interlinked architecture not only makes the modified nanoparticles/layers provide more anchoring sites for chemically binding the polysulfide intermediates, but also provides electronic conduction pathways and works as a mechanical support.^75–77^ Thus, LSBs with modified carbon network electrodes show outstanding performance in terms of sulfur utilization and capacity reversibility. For example, Cui *et al.* reported a sulfur cathode with a polymer modified carbon paper (CP-PVP) current collector (shown in Fig. 9a). The S/CP-PVP (50 μg PVP) cathode showed an initial capacity of 1030 mA h g^−1^ which was just slightly lower than that of S/CP (1100 mA h g^−1^) and a stable capacity of over 900 mA h g^−1^ (87.4%) after 50 cycles, while the S/PVP cathode merely retained a capacity of 200 mA h g^−1^ after 36 cycles (18.2% of the initial capacity).^78^ Zhang *et al.* synthesized titanium-dioxide-grafted carbon paper (CP@TiO_2_) to immobilize sulfur for improving the cycle life of LSBs. As displayed in Fig. 9b, the capacity of the S/CP cathode seriously decreased from 890 to 430 mA h g^−1^ after 200 cycles; conversely, the S/CP@TiO_2_ cathode exhibited an initial discharge specific capacity of 1606 mA h g^−1^ and a reversible capacity of 850 mA h g^−1^ after 200 cycles.^79^ In this work, the initial capacity of the S/CP@TiO_2_ cathode was even larger than that of the S/CP cathode because the charge transfer resistance of the S/CP@TiO_2_ cathode was smaller than that of the S/CP cathode as the electrochemical impedance spectroscopy (EIS) results show. Xu *et al.* prepared S/Co_3_S_4_ on an activated carbon nanofiber (ACNF) cathode (Fig. 9c) which delivered 752 mA h g^−1^ at 2C, whereas the corresponding capacity of the ACNF/S cathode was 430 mA h g^−1^.^80^ After 50 cycles at 1C, the capacity still remained at 610 mA h g^−1^, implying a slow capacity fade of 0.079% per cycle, exhibiting a long-cycle life performance. These studies indicate that various 3D carbon network films modified by metal oxides/sulfides and conducting polymers can effectively improve the cycle performance and guarantee the high utilization of sulfur in a sulfur/carbon cathode, simultaneously.

### 2.2 Ordered array film

In addition to the above-mentioned advantages of self-supporting films, ordered array films produce a number of other benefits:^81–85^ (1) the array structure can provide superior and shorter electron transport routes because the host materials directly grown on the current collector can form ordered electron transport pathways; (2) the array structure with nanostructures aligned on current collectors can lead to uniform distribution of sulfur on host materials, which can further improve the performance of cathodes with large areal sulfur loading; (3) the array structure can form numerous “nano-reservoirs”, and thus can confine polysulfides and minimize the shuttle effect effectively. Therefore, the sulfur cathode using host materials with a binder-free array structure exhibits superior electrochemical performance.

#### 2.2.1 Ordered carbon array structured film

**Fig. 10:**
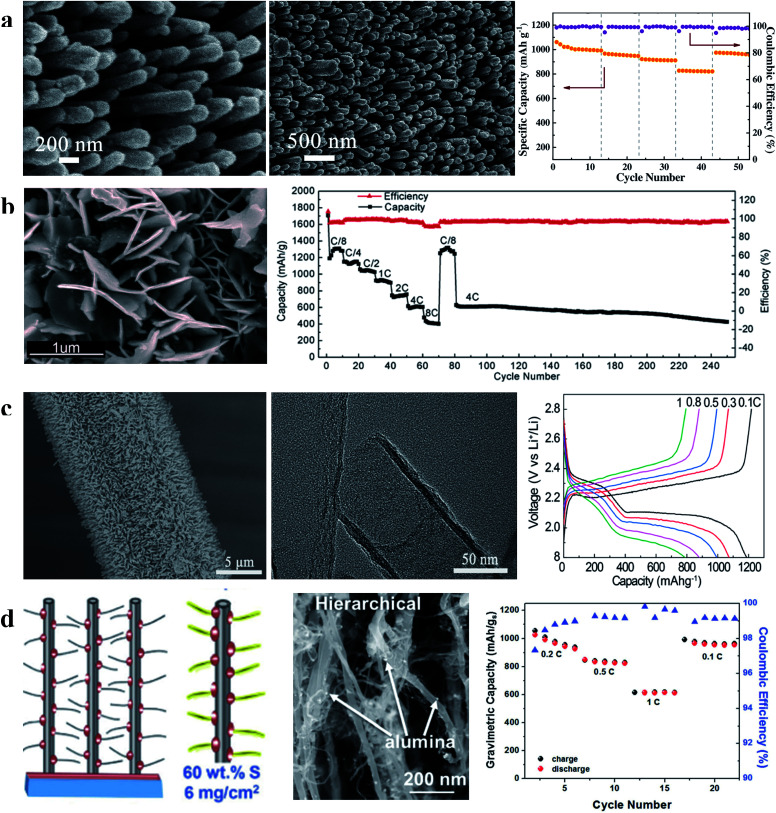
(a) Characterization of the S/CNA cathode and its rate performance; (b) characterization of the S-G NWA cathode and its rate and cycling performances; (c) characterization of CNT arrays on carbon cloth and the first charge–discharge curves of the S/CNT array cathode at different current densities; (d) schematic illustration and characterization of the hierarchical S/CNT array cathode and its rate performance. (a) Reproduced with permission.^86^ Copyright 2016, Elsevier; (b) reproduced with permission.^87^ Copyright 2015, American Chemical Society; (c) reproduced with permission.^88^ Copyright 2016, American Chemical Society; (d) reproduced with permission.^89^ Copyright 2017, Royal Society of Chemistry.

Owing to the advantages of the array structure, sulfur cathodes with carbon array structured films exhibit superior performance compared with cathodes with self-supporting films, especially the rate performance. For example: Liu *et al.* reported a high-performance sulfur cathode with carbon nanorod arrays (CNAs, shown in Fig. 10a), which showed a high capacity of 826 mA h g^−1^ at 2C even when the loading mass of sulfur in the CNA/S composite is about 80.55%, suggesting that the array structure could provide superior and shorter electron transport routes to improve the rate performance of the sulfur cathode, especially when the content of sulfur in the composite is high. The capacity of this cathode can recover to 980 mA h g^−1^ at 0.2C after cycling at a high current rate (2.0C), indicating that shuttling of soluble polysulfides could be blocked by the array structure.^86^ In addition, according to Li *et al.*, the capacity of the cathode with vertically aligned sulfur–graphene nanowall arrays (S-G NWAs, Fig. 10b) was 410 mA h g^−1^ at 8C when the content of sulfur in the composite was as high as 91.5%;^87^ Wu *et al.* reported that a high-performance sulfur cathode could be synthesized by using the CNT array structure (Fig. 10c) and the prepared S/CNT@CC cathode exhibited ∼800 mA h g^−1^ at 1C. These results suggested that the advantages of arrays are present in various carbon materials.^88^ Hierarchical carbon array structured films can further inherit the advantages of binder-free structures because of the more detailed structural design. For example, as Fig. 10d shows, Carter *et al.* designed hierarchical carbon nanotube (CNT) arrays with branches and the sulfur cathode with a high areal loading of 6 mg cm^−2^ prepared using this structure exhibited 655 mA h g^−1^ at 1C.^89^ These studies demonstrate that the carbon array structure can effectively improve the rate performance, even in cathodes with large areal sulfur loading, suggesting that this structure is very suitable for designing cathodes with high power- and energy-density.

**Fig. 11:**
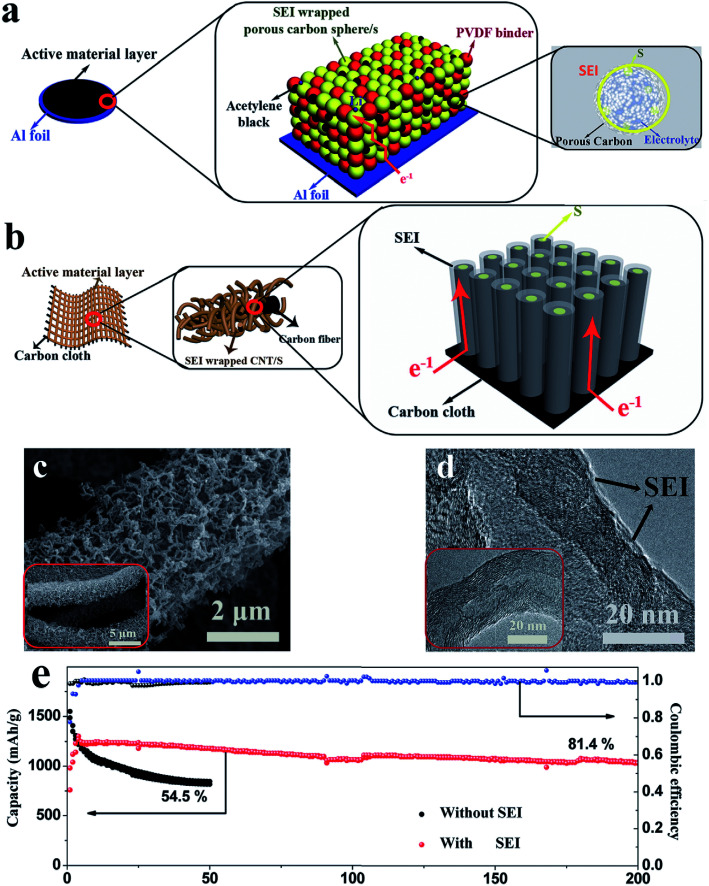
(a) Schematic diagrams of the pathway of electron transport in a powder structured electrode (a) and in an array structured electrode (b); morphology of the SEI wrapped CNT/S array electrode: (c) SEM and (d) TEM; (e) cycle performance of the SEI wrapped CNT/S array electrode at 0.2C. Reproduced with permission.^90^ Copyright 2017, Wiley-VCH.

In addition to the adsorption strategy, recently, an imprisonment strategy has been developed using a facilely formed solid electrolyte interface (SEI) as a smart blocking layer.^90^ This SEI layer can suppress the shuttle effect while allowing lithium ions to pass through, thus improving the cycle stability of sulfur cathodes and ensuring sulfur utilization. Nevertheless, the insulating blocking layer slightly decreases the coulombic efficiency of the electrode (∼95%) because electron transport in the powder electrode will affect the efficiency of this layer (Fig. 11a). This issue can be further solved by using an array structure because such a structure could provide ordered electron transport pathways. As illustrated in Fig. 11b, the cathode with a three-dimensional CNT array structure on carbon cloth (morphology characterization displayed in Fig. 11c and d) allows electron transport from the carbon cloth to the CNTs and sulfur without passing through the SEI layer. As a result, the SEI-wrapped CNT/sulfur array cathode simultaneously demonstrates high coulombic efficiency (∼99%) and good cycle stability (81.4% capacity retention after 200 cycles), indicating that the array structure is very suitable for the imprisonment strategy, as shown in Fig. 11e.

#### 2.2.2 Ordered polar inorganic array structured film

**Fig. 12:**
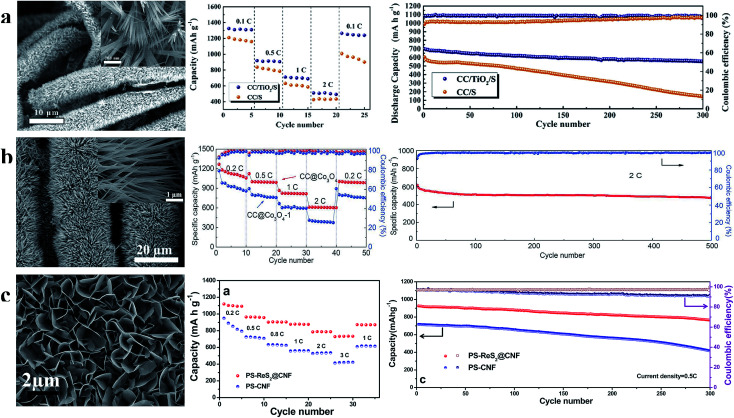
(a) Characterization of the TiO_2_ nanowire array and the rate and cycle performance at 1C of the S/TiO_2_ nanowire array cathode; (b) characterization of the Co_3_O_4_ nanoneedle array and the rate and cycle performance at 2C of the sulfur cathode prepared using the Co_3_O_4_ nanoneedle array; (c) characterization of the ReS_2_ nanosheet array and the rate and cycle performance at 0.5C of the S/ReS_2_ nanosheet array cathode. (a) Reproduced with permission.^95^ Copyright 2018, Elsevier; (b) reproduced with permission.^96^ Copyright 2017, Royal Society of Chemistry; (c) reproduced with permission.^97^ Copyright 2016, American Chemical Society.

Inorganic polar metal oxides can trap polysulfide intermediates more efficiently than carbon materials. However, their application in sulfur cathodes is impeded by the much lower electron conductivity (compared with carbon materials) of polar materials. Therefore, ordered array architectures, which can provide rapid electron and lithium-ion transport, are significantly useful for polar material based sulfur cathodes.^91–94^ Recently, many sulfur cathodes prepared using various polar inorganic arrays without other modifiers exhibited high rate and cycle performance simultaneously, suggesting that ordered array architectures could further promote the development of sulfur/polar inorganic cathodes. According to the literature reported by Yan *et al.* (Fig. 12a), superior rate performance (1325, 918, 710 and 510 mA h g^−1^ at 0.1, 0.5, 1, and 2C, respectively) and ultra-long cycle life (78.4% capacity retention after 300 cycles at 1C and 0.072% per cycle capacity decay) of sulfur cathodes can be realized using the TiO_2_ nanowire array architecture.^95^ As shown in Fig. 12b, Chang *et al.* reported that sulfur cathodes with a Co_3_O_4_ nanoneedle array structure displayed high capacities of 1120, 1010, 830, and 610 mA h g^−1^ at 0.2, 0.5, 1, and 2C, respectively, and a slight capacity decay (0.049% per cycle) at 2C over 500 cycles.^96^ The ReS_2_ nanosheet array also could enable the sulfur cathode to show high performance. The capacities of the S/ReS_2_ nanosheet array cathode (Fig. 12c) were 1100, 960, 903, 876, 787, and 732 mA h g^−1^, and the corresponding current densities were 0.2C, 0.5C, 0.8C, 1C, 2C, and 3C, respectively. The retained capacity after 300 cycles was 750 mA h g^−1^, with only ∼0.063% capacity decay per cycle.^97^

## 3. Binder-free structure for Li-metal anodes

Binder-free films have some unique properties for Li metal anode design, such as a high electrochemical surface area and high electric conductivity, which can decrease the effective current density for Li-metal deposition/dissolution.^98–100^ Moreover, with a suitable porous structure, binder-free films can accumulate Li deposits within its internal space, thus minimizing the possibility of undesired dendritic growth of Li-metal.^101–103^ Therefore, it is well recognized that lithium metal electrodes with a self-standing film host structure has practical values for future applications.^104–106^

### 3.1 Three-dimensional conductive material (carbon, metal) network binder-free film

**Fig. 13:**
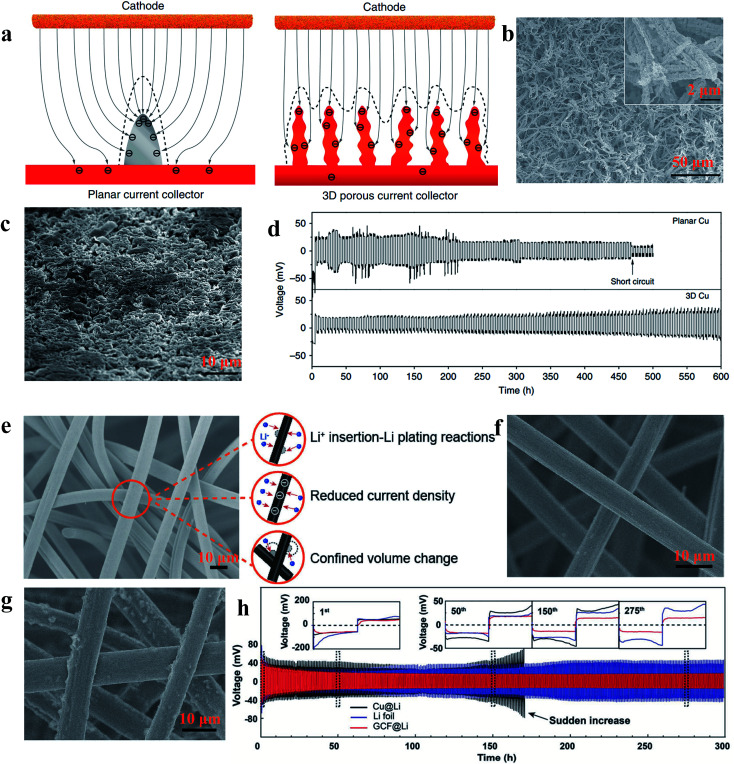
(a) Illustration of the proposed electrochemical deposition processes of Li metal on the planar current collector and 3D current collector; SEM images of 3D Cu foil (b) and 3D Cu foil with 2 mA h cm^−2^ of Li loading (c); (d) voltage profiles of Li metal plating/stripping at 0.2 mA h cm^−2^ on different current collectors; (e) illustration of the versatility of the 3D GCF structure; SEM images of 3D GCF (f) and 3D GCF with 8 mA h cm^−2^ of Li loading (g); (h) galvanostatic plating/stripping profiles on different current collectors. (a–d) Reproduced with permission.^107^ Copyright 2015, Nature Publishing Group; (e and f) Reproduced with permission.^108^ Copyright 2017, Wiley-VCH.

According to the work reported by Yang *et al.*, the 3D current collector can optimize the deposition behaviour of Li significantly due to its submicron skeleton and high electroactive surface area, thus restricting the formation of Li dendrites. As shown in Fig. 13a, the submicron skeleton of 3D Cu could make the electric field uniform because the submicron skeleton of the 3D Cu foil can adjust the charge centers and nucleation sites, leading to homogeneous dispersion of Li (Fig. 13b and c). With the advantages of a 3D current collector, the Li anode designed in this work could be stably cycled without short circuit and large voltage hysteresis for 600 h (Fig. 13d).^107^ Zuo *et al.* showed that multifunctional 3D current collectors (Fig. 13e) could effectively improve the electrochemical performance due to the following advantages: (1) the interlinked carbon fibers with large surface area can lower the local current density, giving rise to more uniform electrochemical deposition of Li; (2) the porous structure of the 3D current collector has enough space to mitigate Li plating/stripping processes. The Li anode with this versatile 3D current collector in this work displayed an areal capacity as high as 8 mA h cm^−2^ without obvious dendrite formation (as Fig. 13f and g shows) and ran for 300 h without large voltage hysteresis (Fig. 13h).^108^ Therefore, various types of 3D network electrodes with different carbon/metal materials have been reported recently, including carbon materials,^109,110^ Cu^111,112^ and Ni,^113^ which have been employed as host frameworks to accommodate lithium storage.

### 3.2 Three-dimensional hybrid material network binder-free film

**Fig. 14:**
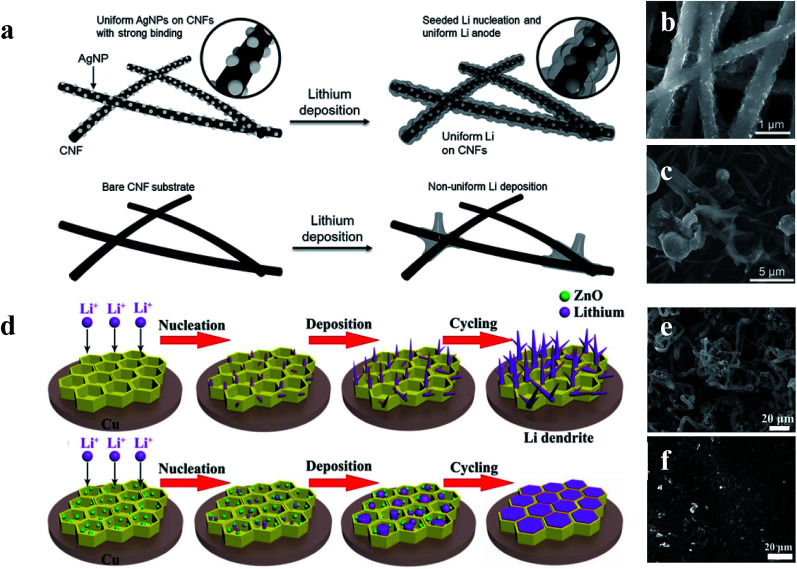
(a) Schematic diagrams of the comparison between Li deposition on CNFs and CNFs with AgNPs; SEM images of 1 mA h cm^−2^ of Li deposited on CNFs with AgNPs (b) and CNFs (c); (d) schematic diagrams of the comparison between Li deposition within the HCP scaffold with and without the decoration of ZnO nanoparticles; SEM images of HCP (e) and ZnO@HPC electrodes (f) after 80 cycles at 1 mA cm^−2^ with a Li stripping/plating capacity of 1 mA h cm^−2^. (a–c) Reproduced with permission.^121^ Copyright 2017, Wiley-VCH; (d–f) reproduced with permission.^122^ Copyright 2017, Elsevier.

Although the Li anodes with a 3D conductive material network architecture displayed superior electrochemical performance in many previous studies, the Li wettability on the surface of hosts still restricts the further improvement of Li anodes.^114–116^ To address this issue, surface-modified 3D host material network structures within lithiophilic materials have been developed to enhance the wetting properties between metallic lithium and host materials, leading to superior electrochemical performances during lithium plating/stripping.^117–120^ Yang *et al.* utilized silver nanoparticle (AgNP) modified 3D carbon nanofibers (CNFs) to impede the formation of Li dendrites. As shown in Fig. 14a, since Ag has an appreciable solubility in Li according to the Li–Ag phase diagram, Ag nanoseeds could guide more uniform deposition of Li on CNFs, which can be confirmed by the SEM comparison of 1 mA h cm^−2^ of Li deposited on CNFs with AgNPs (Fig. 14b) and CNFs (Fig. 14c).^121^ According to Jin *et al.*, a ZnO modified 3D hierarchical porous carbon (HCP) scaffold also could lead to more uniform deposition of Li (Fig. 14d). This is because Li ions prefer to react with ZnO seeds (Li^+^ + ZnO → Li_2_O + Zn) during the initial process, thus resulting in the formation of Zn, which is soluble in Li according to the phase diagrams. Therefore, the existence of Zn induced the promising deposition of Li on the 3D HCP, leading to a dendrite-free Li metal anode (Fig. 14e and f).^122^

**Fig. 15:**
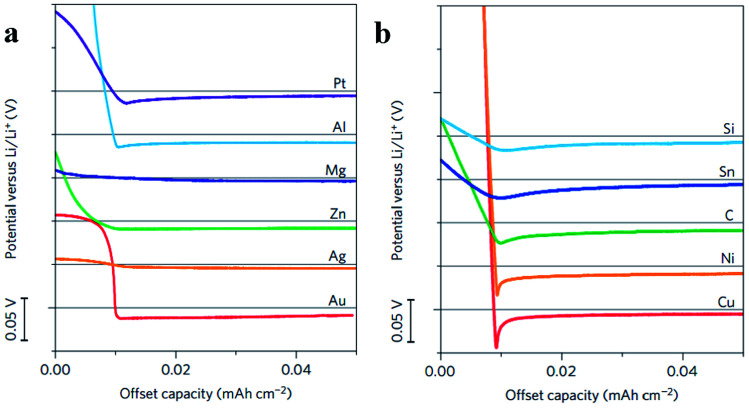
(a) Voltage profiles of various materials with some solubility in Li during Li deposition at a current density of 10 μA cm^−2^; (b) shifted voltage profiles of various materials with negligible solubility in Li during Li deposition at a current density of 10 μA cm^−2^; the horizontal gray lines show 0 V *versus* Li in (a) and (b). (a–c) Reproduced with permission.^123^ Copyright 2016, Nature Publishing Group.

To find the materials which can enhance the wetting properties between metallic lithium and host materials, Yan *et al.* did a very meaningful study which measured the overpotential during Li deposition on various substrates. The results are provided in Fig. 15a and b. This study confirmed that Li metal nucleation overpotential on materials with definite solubility (Pt, Al, Mg, Zn, Ag and Au) in Li was essentially zero, leading to preferential Li metal deposition. Materials showing no solubility (Cu, Ni, C, Sn, and Si) in lithium were also tested, and the results suggested that all five materials show a clear overpotential for Li metal nucleation, and form compound alloy phases with Li. Therefore, according to these studies, the selective deposition of Li through heterogeneous seeded growth can be realized, which is significant for further modification of binder-free structured Li metal anodes.^123^

**Fig. 16:**
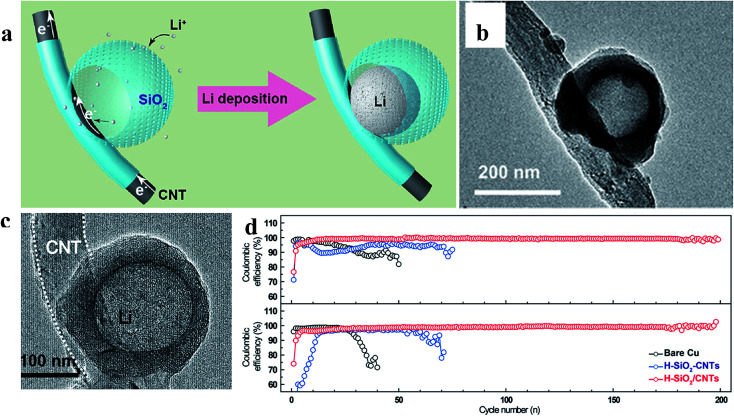
(a) Illustration of the proposed electrochemical deposition processes of Li metal on HSMS with a CNT core architecture; TEM images of HSMS with a CNT core structure (b) and the structure after Li deposition (c); (d) cycle performance of Li metal plating/stripping at 0.2 and 0.5 mA cm^−2^ with a total capacity of 2 mA h cm^−2^. (a–d) Reproduced with permission.^124^ Copyright 2017, American Chemical Society.

**Fig. 17:**
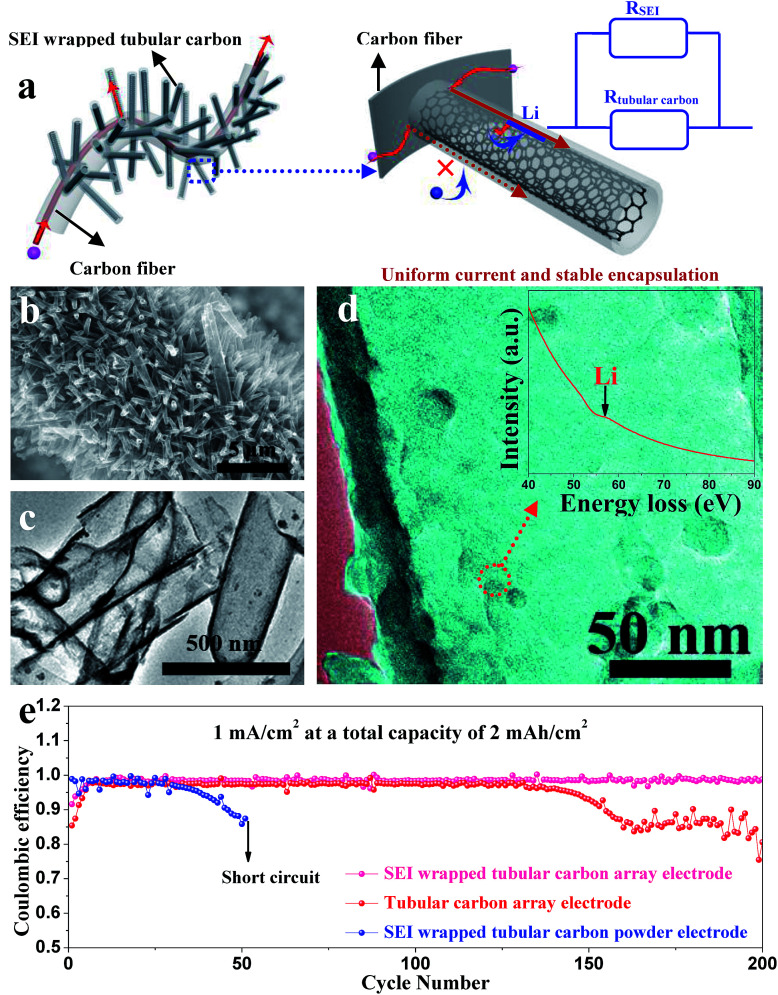
(a) Illustration of electron transport pathways and Li plating in the SEI wrapped TCA electrode; (b) SEM image and (c) TEM image of the TCA structure; (d) TEM image of tubular carbon in the TCA electrode after cycling; the inset is the corresponding EELS spectra; (e) coulombic efficiencies of Li metal plating/stripping in different electrodes at 1 mA cm^−2^ with a total capacity of 2 mA h cm^−2^. (a–d) Reproduced with permission.^125^ Copyright 2019, Royal Society of Chemistry.

Zuo *et al.* provided an alternative way to address this issue, *via* hollow silica microspheres (HSMS) with a carbon nanotube (CNT) core architecture (Fig. 16). In this structure, the insulating coating layer prevents Li ions from acquiring electrons on the CNTs and thus guiding the selective deposition of Li in HSMS, giving rise to dendrite-free growth (Fig. 16a–c). The anode designed using this strategy maintained a high coulombic efficiency during 300 cycles even at a current density of 0.2 mA cm^−2^ or a higher one of 0.5 mA cm^−2^ with a total capacity of 2 mA h cm^−2^ (Fig. 16d).^124^ However, there is no ordered transmission path for electrons in the powder electrode, leading to complex or defective control of electrons. Therefore, the array structure which can optimize the transmission path of electrons has a very broad application prospect in this strategy. For example, Guo *et al.* designed an SEI wrapped tubular carbon array (TCA) structure to improve the performance of lithium metal anodes *via* an electron regulation strategy (Fig. 17a–c). In this structure, electrons can be transported directly to tubular carbon without passing through the SEI, thus guiding selective Li deposition on the inner-surface of tubular carbon, leading to dendrite-free growth (Fig. 17d). The anode designed using this strategy (SEI wrapped TCA electrode) maintained a higher and more stable coulombic efficiency during 200 cycles compared with the electrode without SEI wrapping (TCA electrode) or the powder electrode (TCP electrode), indicating that the array structure is very helpful.^125^

## 4. Conclusions and outlook

In conclusion, the recent advances in design and fabrication of binder-free architectures as state-of-the-art electrodes for LSBs have been summarized and the following conclusions can be drawn:

(1) Although both the binder-free 3D self-standing film and array film can improve the performance of LSBs, there are different priorities of their functions. For example, the array film is particularly suitable for addressing the issue of sulfur cathodes with polar metal oxides and sulfides, since the array structure can provide better electron transport paths compared with the 3D self-supporting structure. Compared with the strategy of compositing with the electric conduction materials, this method is not only simple, but also does not increase the amount of inactive materials which is essential for the energy density of the whole cathode. Similarly, the array structure can make the current distribution more uniform than the 3D self-standing film because of the better electron transport paths in array films, and thus effectively inhibiting the formation of lithium dendrites. However, large-scale preparation of array films is more difficult than that of self-standing films, which without the substrate also can make the sulfur cathode or Li anode maintain larger energy density.

(2) Although sufficient space between materials in binder-free films can effectively accommodate the high volume expansion of sulfur/lithium, these films with an open architecture, especially array structure films, fail to imprison sulfur/lithium significantly compared with electrodes with closed structures (like hollow spheres).

(3) The method to inhibit dendrite formation *via* controlling Li ions receiving electrons at a specified place is a very significant new direction for designing Li metal anodes. However, there is no ordered transmission path for electrons in the powder electrode, leading to a complex or defective regulation of electron transmission. Array structures can provide very ordered transport pathways, leading to easier regulation of electron transmission. Therefore, the array structure has a very broad application prospect in this strategy. However, the carbon materials used in existing studies with low Li wettability restrict the further improvement of Li anodes.

Therefore, although some significant progress has been made, several possible future prospects, which have not received sufficient attention and research, can be drawn from the above conclusions such as:

(1) Large-scale preparation of array structures: although the array structures can be facilely synthesized by using an anodized aluminium oxide (AAO) template, there is still a demand for simpler and more economical ways to prepare array structures on a large scale. For example, with appropriate electric or magnetic fields, array architectures may be prepared by the casting preparation method of the powder electrode.

(2) Encapsulation of the array structure: for example, a porous film formed on the top of array structures can make the “reservoir-like” array structure become a “house-like” array structure. With this “house-like” array structure, better imprisonment of sulfur/lithium loading in the gaps can be realized, suggesting that films with encapsulation of the array structure can accommodate higher sulfur/lithium loading. It should be noted that the formation of this film must be simple, preferably in one step, to ensure the applicability of the method.

(3) The array structure with lithiophilic materials: to further develop the electron regulation strategy, the carbon array structure should be replaced by cheap lithiophilic materials with high electrical conductivity: (1) the cheap materials can ensure the economy of this strategy; (2) the high conductivity of materials (compared with the coating layer) can guarantee that electrons merely pass along the inner surface of the array structure; (3) the Li wettability of materials can lead to easier deposition of Li metal.

## Conflicts of interest

There are no conflicts to declare.

## Supplementary Material
